# Risk of Suicide in Patients With Traumatic Injuries

**DOI:** 10.1001/jamanetworkopen.2025.54168

**Published:** 2026-01-15

**Authors:** Anders Rasmussen, Trond Nordseth, Jo Steinson Stenehjem, Jon Michael Gran, Lars Lien, Leiv Arne Rosseland

**Affiliations:** 1 Institute of Clinical Medicine, Faculty of Medicine, University of Oslo, Oslo, Norway; 2 Department of Anesthesia and Intensive Care Medicine. Innlandet Hospital Trust, Hamar/Elverum, Norway; 3 Division of Prehospital Medicine, Innlandet Hospital Trust, Hamar, Norway; 4 Department of Research and Development, Division of Emergencies and Critical Care, Oslo University Hospital, Oslo, Norway; 5 Department of Anesthesia and Intensive Care Medicine, St Olav Hospital, Trondheim, Norway; 6 Department of Circulation and Medical Imaging, Norwegian University of Science and Technology, Trondheim, Norway; 7 Møre and Romsdal Hospital Trust, Ålesund, Norway; 8 Department of Biostatistics, Oslo Centre for Biostatistics and Epidemiology, University of Oslo, Oslo, Norway; 9 Department of Research, Cancer Registry of Norway, Norwegian Institute of Public Health, Oslo, Norway; 10 Inland Norway University of Applied Sciences, Elverum, Norway

## Abstract

**Importance:**

Previous studies suggest that critically injured patients are at increased risk of suicide following discharge, but these have mainly been single-center studies or had limited data on comorbidities and socioeconomic factors.

**Objective:**

To examine the risk of suicide after hospitalization for traumatic injuries.

**Design, Setting, and Participants:**

This nationwide register-based cohort study used data from 5 Norwegian nationwide health registries and Statistics Norway between 2014 and 2020. Patients registered in the Norwegian Trauma Registry (NTR) for whom trauma team activation at hospital arrival was mandated by national guidelines between 2015 and 2018 were matched to general-population controls on gender and birth year in a 1:10 ratio according to a matched cohort design. Patients discharged alive were followed-up for a minimum of 2 years. All Norwegian hospitals treating patients with traumatic injuries provide data to the NTR. The final analysis was conducted in April 2025.

**Exposures:**

Traumatic injury, admitted to hospital, and registered in the NTR.

**Main Outcomes and Measures:**

The outcome of interest was suicide, as registered in the Norwegian Cause of Death Registry. Cumulative incidence ratios (CIR) of suicide with 95% CIs, taking nonsuicidal death as a competing event into account, were estimated. Adjustments for Charlson Comorbidity Index, previous psychiatric illness, and socioeconomic position (SEP) were conducted using inverse probability of treatment weights.

**Results:**

A total of 25 536 patients with traumatic injuries (165 897 [67%] male; mean [SD] age, 41 [23] years) were matched to 247 095 controls, with a mean (SD) age of 41 (23) years and 68% male. The cumulative incidences of suicide were 0.18% at 2 years and 0.34% and 5 years for patients with traumatic injuries and 0.02% at 2 years and 0.05% at 5 years for controls (2-year CIR, 9.3 [95% CI, 5.4-13.0]; 5-year CIR, 6.9 [95% CI, 4.4-9.1]). Patients with traumatic injury were older at the age of suicide compared with controls (mean [SD] age, 43 [19] years vs 36 [17] years; *P* = .03) and female patients with traumatic injury had higher incidence of suicide compared with female controls (36% vs 17%; *P* = .005).

**Conclusions and Relevance:**

In this cohort study of patients in Norway discharged alive after critical injury, a 9-fold increased risk of suicide after 2 years was observed. These findings suggest that follow-up is warranted for possible psychological distress in this patient group.

## Key Points

**Question:**

Is there an increased risk of suicide for patients admitted to the hospital for traumatic injuries compared with general-population controls?

**Findings:**

In this cohort study including 25 536 patients from the Norwegian Trauma Registry matched with 247 095 controls, risk of suicide was increased 9-fold among survivors of traumatic injury at 2 years after index injury vs controls.

**Meaning:**

The findings of this study suggest an increased risk of suicide among patients who survive traumatic injury, which may necessitate psychological follow-up among this group.

## Teaser

This cohort study examines risk of suicide among patients hospitalized for traumatic injuries compared with general population controls, with up to 5 years of follow-up.

## Introduction

According to the World Health Organization, more than 720 000 people die from suicide each year globally. Suicide is considered the third leading cause of death among adolescents and young adults.^1^ In 2023, 693 people died of suicide in Norway, of whom 71% were males.^2^ Psychiatric illness is considered a major risk factor for suicide, and traumatic injuries may exaggerate these.^3^ Finstad et al^4^ reported that survivors of severe injury had feelings of insecurity, pain, and mental health problems after discharge. Haider et al^5^ reported that 20% of patients with traumatic injuries were diagnosed with posttraumatic stress disorder, an important risk factor for suicide.

In 2003, Conner et al^6^ published a study from New Zealand that reported increased relative risk (RR) of self-injury and suicide following traumatic injury. The study lacked reporting on measures of socioeconomic position (SEP), previous psychiatric disease, and comorbidities. In a single-center trial from 2005, Ryb et al^7^ found that suicide was more common among survivors of traumatic injury compared with the general population. Neither of these studies adjusted for comorbidities, previous mental health problems, or SEP.^6,7^ A Canadian matched-cohort study including 2253 patients with traumatic injuries with severe or very severe injuries reported an increased risk of suicidality in patients with critical injuries vs controls.^8^ Nationwide register-based studies in patients with traumatic injuries with high-quality data on trauma, pretrauma mental health, comorbidities, and SEP are lacking. To inform the public and policymakers, studies on suicide and its risk factors from high-quality registry data are warranted.

This Norwegian nationwide register-based study aimed to examine the hypothesis that patients are at increased risk of suicide after injury. We also aimed to assess how injury severity, type of injury, pretrauma mental health, and socioeconomic factors are associated with risk of suicide after hospital discharge.

## Methods

This is a matched cohort study of patients registered in the Norwegian Trauma Registry (NTR) between January 1, 2015, and December 31, 2018. The study was approved by the regional committee for medical research ethics in South-Eastern Norway and the data protection officer at Oslo University Hospital. The ethical committee approved a waiver of informed consent per the Health Research Act. Patients were matched on gender and birth year with 10 controls from the Norwegian Population Registry at the date of injury (index date).^9^ The study is part of the Injury Prevention and Outcomes Following Trauma (IPOT) research project at Oslo University Hospital. In reporting the study, we followed the Strengthening the Reporting of Observational Studies in Epidemiology (STROBE) reporting guideline on cohort studies.

### Population

In Norway, inhabitants are identified by a personal identification number used when they are in contact with the health care system. The Norwegian health care system is publicly funded. The trauma system includes 4 major regional trauma centers (Oslo, Trondheim, Tromsø, and Bergen) and 34 acute care trauma hospitals. Since 2015, it has been mandatory for all Norwegian hospitals to report data on traumatic injuries to the NTR.^10^ Specially trained registrars at each location ensure high data quality.^11^ A national guideline on how to assess vital signs, anatomical injuries, and the mechanism of injury guides the decision for trauma team activation (TTA).^12^ All patients in whom TTA is undertaken are registered in the NTR. If the registrar finds that TTA was not activated in hospitalized patients with New Injury Severity Score (NISS) greater than 12 or other signs of severe injury, the patients are registered in the NTR. To reflect the general Norwegian population, the control group was drawn from the National Population Register. Sampling was conducted with replacement to allow for both a matched cohort study and a nested case-control design, resulting in some individuals being controls for more than 1 patient. Only unique controls were included in this analysis, resulting in a mean (SD) of 9.68 (0.60) controls per case. The included controls had to be alive at the index date, residing in Norway, and not registered in the NTR during the observational period.^9^

**Figure 1.**
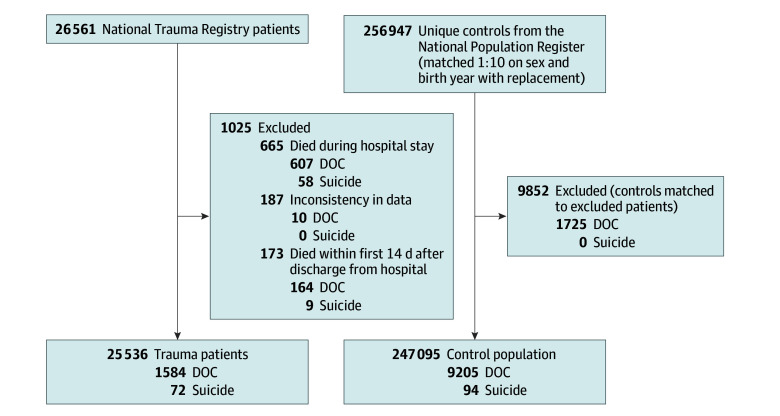
Overview of the Study Population Controls were matched to trauma cases on their injury date based on gender and birth year. DOC indicates death of other cause (ie, not suicide).

A total of 26 561 patients with traumatic injuries registered in the NTR in the study period were assessed for inclusion, matched to 256 907 unique controls.^9^ We excluded 187 patients with traumatic injuries with inconsistent data. Patients with traumatic injuries who died during hospital stay (647 patients) or within 2 weeks after discharge (173 patients) were excluded. Controls for these excluded patients were also excluded (9852 controls). An overview of the study population is presented in Figure 1.

### Data Sources

Data on study participants were collected between 2014 and 2020, yielding at least 1 year of data prior to injury and a minimum of 2 years after injury. Data were collected from the Norwegian Patient Registry (NPR), the Norwegian Prescription Database (NorPD), the Norwegian Control and Payment of Health Reimbursement Database (KUHR), the Norwegian Cause of Death Registry (NCDR), and Statistics Norway. The NPR data contained information on all health care encounters with hospitals and other specialized health care, including the time of contact and *International Statistical Classification of Diseases and Related Health Problems, Tenth Revision* (*ICD-10*) codes. The Charlson Comorbidity Index (CCI) was provided by the NPR on all study participants as an assessment of comorbidity and calculated at the end of follow-up. The NorPD data contained information on all medications dispensed in the study period, including time of dispensing and the *ICD-10* or International Classification of Primary Care (ICPC-2) diagnoses provided on governmentally reimbursed prescriptions. All contacts with primary care physicians and other types of primary care practitioners were registered in the KUHR data, including time of contact and *ICD-10* or ICPC-2 diagnosis. The NCDR provided data on causes of death. Data on income, educational level, marital status, and date of death were provided by Statistics Norway. A study protocol with a comprehensive description of the IPOT study has previously been published,^9^ as have 2 reports using the same cohort.^13,14^

### Measures

NTR provided data on age, gender, anatomic injury, and cause of injury. Injuries were classified according to the 2008 update of the Abbreviated Injury Scale (AIS). Injury Severity Score (ISS) and NISS were calculated from the AIS codes. To illustrate associations and risk factors between injury as exposure and suicide as outcome used in the inverse probability of treatment weights (IPTW) analysis, a directed acyclic graph (DAG) was constructed (eFigure 1 in Supplement 1).^15^

Study participants were classified as having a preexisting psychiatric diagnosis if they had 1 or more health care contacts or dispensed medications reimbursed with *ICD-10* diagnoses chapter F and/or ICPC-2 diagnoses chapter P registered in the NPR, KUHR, or NorPD before the index date. Posttraumatic psychiatric diagnosis was defined as 1 or more such contacts registered after hospital discharge. SEP was assessed based on educational level, marital status, and income.^16^ Educational level was categorized as completed elementary and middle school, high school, vocational training, or college or university.^16^ When stratifying the educational levels, a separate category for study participants younger than age of 20 years on educational level was defined. Income level was categorized into 4 groups, applying the 2017 exchange rate with Norwegian krone, as low, less than US $37 000; lower middle, US $37 000 to US $67 000; upper middle, US $68 000 to US $97 000; and high, more than US $97 000.

### Outcome

The primary outcome was death by suicide after hospital discharge, taking death of other cause (DOC) as a competing event into account. Patients who were admitted due to a suicide attempt, died during hospital stay, or who died of suicide shortly after discharge were excluded. The start of follow-up was set at 2 weeks after hospital discharge. This was done to prevent patients who were initially admitted as patients with traumatic injuries after a suicide attempt being misclassified as suicide after trauma and to take possible inconsistencies in dates of admittance and discharge into account when estimating the risk of suicide after discharge. The controls were followed-up from the index date registered for the matched case, as the date of discharge for the patient did not give any meaningful information with respect to follow-up of the primary outcome for controls.

### Statistical Analysis

Event times were calculated as time from start of follow-up to suicide, DOC, or censoring, whichever occurred first. For patients with traumatic injuries, only follow-up after the first recorded trauma was done and later trauma events were not considered in the analysis. Censoring was performed at the end of the follow-up period (December 31, 2020).

The cumulative incidences of suicide and the competing event of DOC following discharge were estimated using the Aalen-Johansen estimator.^17^ Cumulative incidence ratios (CIRs) are presented with 95% CIs. To account for potential confounding from pretrauma psychiatric diagnoses, comorbidities and SEP, stabilized IPTW were used to generate adjusted cumulative incidence curves.^18^ The weights were constructed using logistic regression, estimating each individual’s probability of have a traumatic injury. CIs were estimated using nonparametric bootstraps. As the number of missing data points were low, a complete-case analysis was conducted. Statistical analysis was done in R software version 4.2.3 (R Project for Statistical Computing). The R packages tidyverse, gt-summary, ggsurvfit, survival, and boot were applied in the data management and statistical analysis.^19,20,21,22,23^ *P* values were 2-sided, and statistical significance was set at *P* ≤ .05. The final analysis of data was conducted in April 2025.

## Results

This study included 25 653 patients with traumatic injuries matched to 247 095, with a mean (SD) age of 40 (23) years and 68% males. The main mechanisms of trauma were traffic incidents and falls. Most of patients (16 113 patients [63%]) had minor trauma (ISS, 1-8), 2140 patients (8%) had severe trauma, and 1256 patients (5%) had very severe trauma. Baseline demographic data are presented in the Table.

**Table.** Baseline Demographic Data for the Study Population

Characteristic	Individuals, No. (%)		*P* value^a^
	Control (n = 247 095)	With traumatic injuries (n = 25 536)	
Gender			
Female	81 198 (33)	8292 (32)	NA
Male	165 897 (67)	17 244 (68)	
Age, mean (SD), y	41 (23)	41 (23)	NA
Suicide deaths	94 (0.04)	72 (0.28)	<.001
Charlson Comorbidity Index			
0	186 160 (75)	16 860 (66)	<.001
1	28 483 (12)	3976 (16)	
2	15 665 (6)	2154 (8)	
3	6949 (3)	1023 (4)	
4	3333 (1)	559 (2)	
5	1667 (1)	300 (1)	
≥6	4838 (2)	664 (3)	
Educational level			
Age <20 y	55 640 (23)	5755 (23)	<.001
Elementary or middle school	45 095 (19)	6510 (26)	
High School	22 384 (9)	2375 (10)	
Vocational training	60 882 (25)	5953 (24)	
College or university	56 145 (23)	4437 (18)	
Missing data, No.	6949	506	
Income, US $			
<37 000	114 969 (49)	13 056 (52)	<.001
37 000-67 000	70 411 (30)	7374 (30)	
68 000-97 000	30 625 (13)	2798 (11)	
>97 000	20 490 (9)	1732 (7)	
Missing data, No.	10 600	576	
Marital status			
Divorced or separated	20 489 (8)	2955 (12)	<.001
Married or widowed	92 864 (38)	8243 (32)	
Unmarried	126 366 (51)	14 087 (55)	
Missing data, No.	7376 (3)	251 (1)	
Any preindex *ICD-10* F-diagnosis or ICPC-2 P-diagnosis	60 755 (25)	9751 (38)	<.001
Any preindex *ICD-10* F-diagnosis	19 572 (8)	4660 (18)	<.001
Any postindex *ICD-10* F-diagnosis or ICPC-2 P-diagnosis	78 275 (32)	12 869 (50)	<.001
Mechanism of injury			
Traffic collision	NA	11 770 (47)	NA
Violence	NA	3434 (14)	NA
Falls	NA	8930 (36)	NA
Other mechanisms	NA	750 (3)	NA
Missing data, No.	NA	652	NA
Injury Severity Score category (score)			
Minor (1-8)	NA	16 113 (63)	NA
Moderate (9-15)	NA	6027 (24)	NA
Severe (16-24)	NA	2140 (8)	NA
Very severe (≥25)	NA	1256 (5)	NA

Abbreviations: *ICD-10*, *International Statistical Classification of Diseases and Related Health Problems, Tenth Revision*; ICPC-2, International Classification of Primary Care; NA, not applicable.

^a^ Pearson χ^2^ test; Welch 2-sample *t* test.

**Figure 2.**
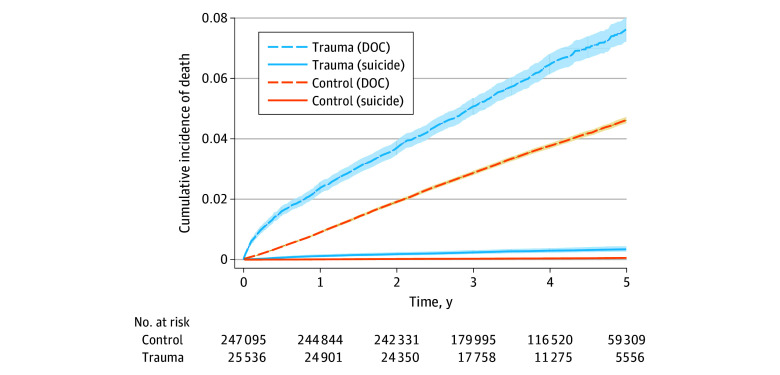
Cumulative Incidence of Suicide and Death of Other Causes (DOC) Aalen-Johansen estimates for cases and controls included in this matched cohort study. Shading indicates 95% CIs.

**Figure 3.**
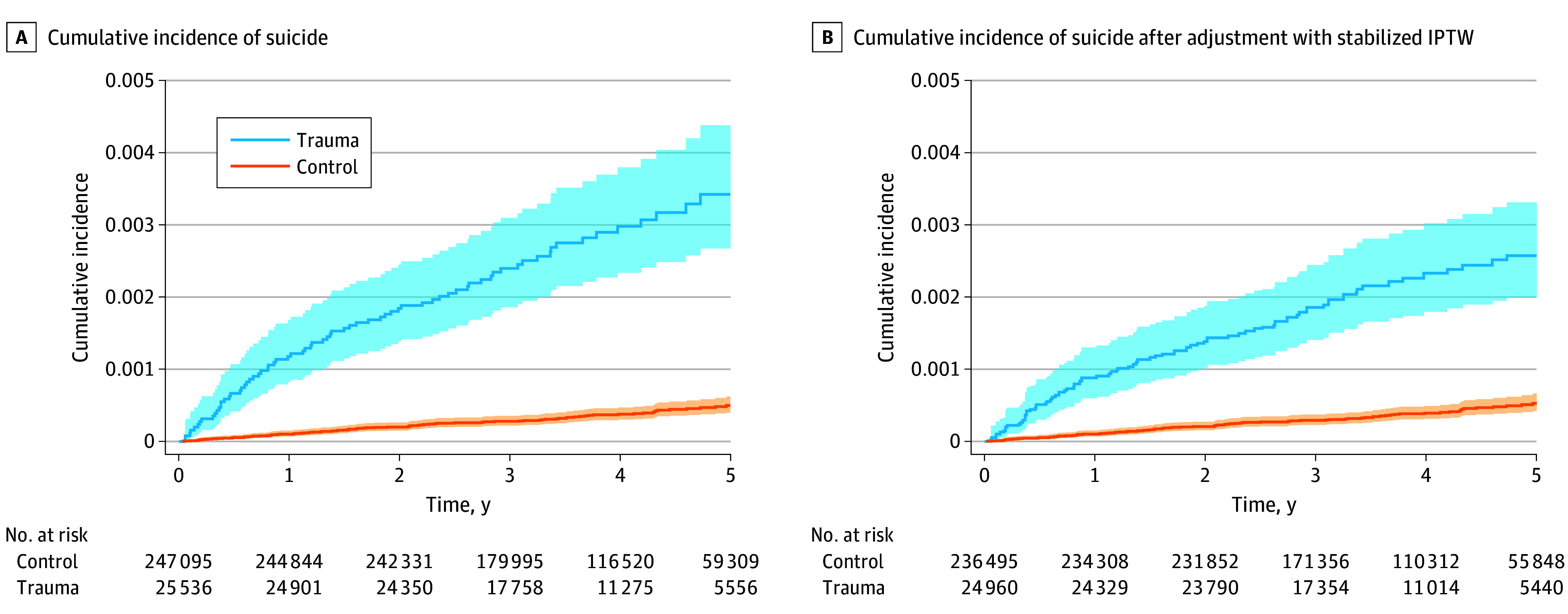
Cumulative Incidence of Suicide Among Patients With Traumatic Injuries and Controls A, Controls were matched on gender and birth year. B, Adjusted for comorbidity, income, education, marital status, and previous psychiatric illness. Curves for the competing event death of other cause are not plotted but taken into account in the analysis. IPTW indicates inverse probability of treatment weights; shading, 95% CIs.

**Figure 4.**
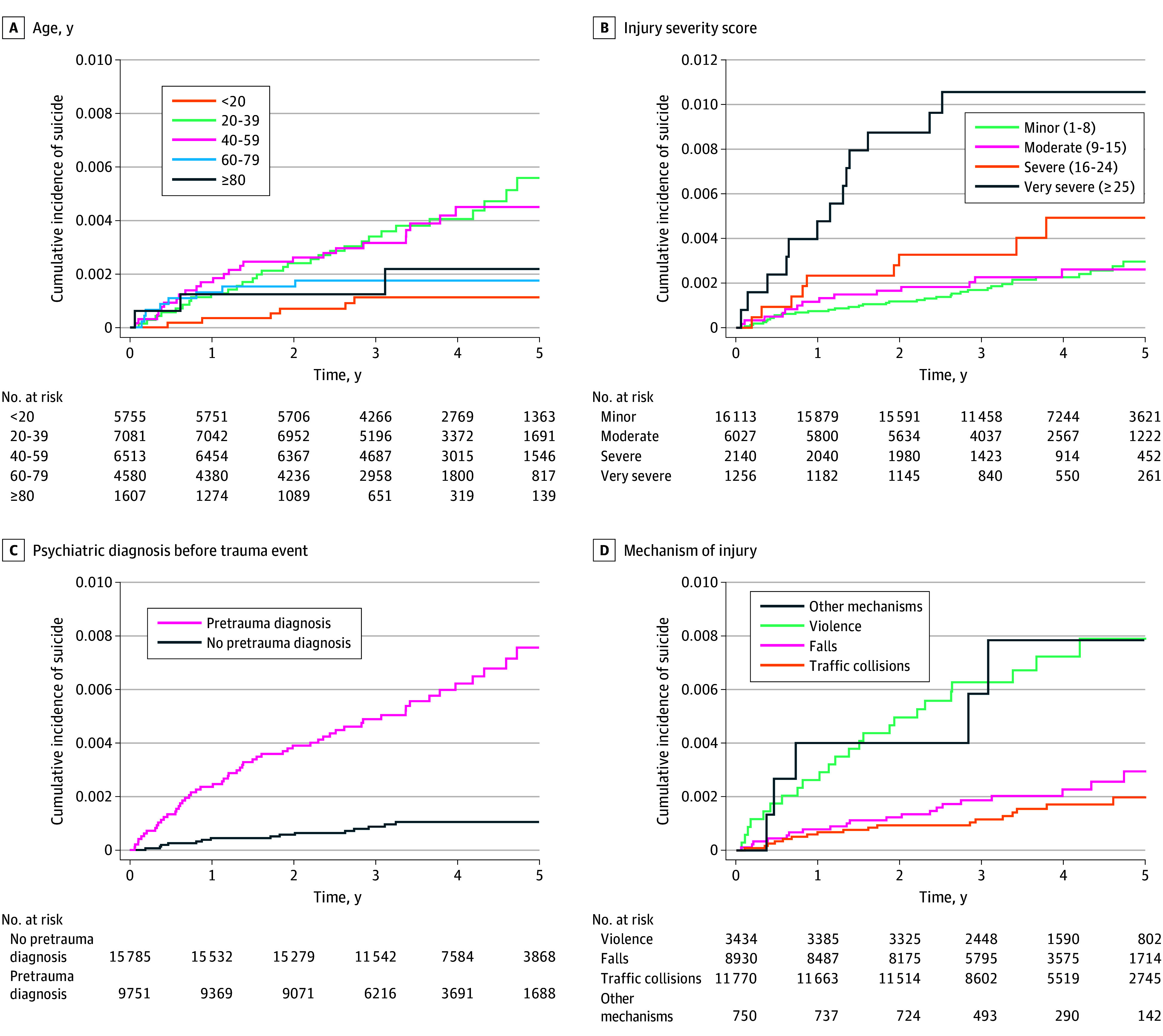
Univariate Estimates of Cumulative Incidences of Suicide Among Patients With Traumatic Injuries

Suicide occurred among 72 patients with traumatic injuries (0.28%) and 94 controls (0.04%) in the study period (*P* < .001), of which 52 and 49 deaths occurred at 2 years after start of follow-up, respectively. Among injured individuals who died of suicide, 9 patients (12%) had severe trauma (ISS, 16-24) and 13 patients (18%) had very severe trauma (ISS, >24). Violence as a mechanism of injury was observed in 24 patients (36%) who died of suicide, whereas 3434 patients (14%) in the overall traumatic injury cohort had violence as a mechanism of injury. Previously registered psychiatric diagnoses and psychiatric diagnoses registered after discharge were more prevalent among patients who died of suicide compared with patients without suicide (eTable 1 in Supplement 1). The unadjusted cumulative incidences of suicide for patients and controls, taking DOC as a competing event into account, are presented in Figure 2. The cumulative incidences of suicide were 0.18% at 2 years and 0.34% at 5 years among patients with traumatic injuries and 0.02% at 2 years and 0.05% at 5 years among controls (2-year CIR, 9.3 [95% CI, 5.4-13.0]; 5-year CIR, 6.9 [95% CI, 4.4-9.1]). Figure 3A illustrates the unadjusted cumulative incidence of suicide, whereas the stabilized IPTW-adjusted cumulative incidence curves are illustrated in Figure 3B. In the IPTW-adjusted model, the cumulative incidences of suicide were 0.14% at 2 years and 0.26% at 5 years among patients with traumatic injuries, and 0.02% at 2 years and 0.05% at 5 years among controls (2-year CIR, 6.8 [95% CI, 3.6-9.6]; 5-year CIR, 4.9 [95% CI, 3.1-6.5]). Figure 4 illustrates the cumulative incidence of suicide for patients stratified by age group, registered pretrauma psychiatric diagnosis, ISS, and mechanism of trauma. The corresponding associations of gender, posttrauma psychiatric diagnosis, marital status, income, and level of education are illustrated in eFigure 2 in Supplement 1.

Patients aged between 20 and 59 years had an increased cumulative incidence of suicide compared with other age groups. The severity of trauma was associated with increased the incidence of suicide, with a higher risk among patients with ISS greater than 15. Violence as the mechanism of injury was associated with increased risk of suicide. When comparing patients and controls who died of suicide, the age at time of suicide was higher in the trauma population (mean [SD] age, 43 [19] years vs 36 [17] years; *P* = .03). The proportion of females who died suicide also differed between groups (36% in the traumatic injury group vs 17% in the control group; *P* = .005), patients with traumatic injuries who died of suicide were more likely to have a known psychiatric diagnosis before index date compared to controls (79% vs 64%; *P* = .03). They were also more likely to have psychiatric health care contacts following injury compared to controls (92% vs 80%; *P* = .03). Other relevant differences between patients and controls who died of suicide are presented in eTable 2 in Supplement 1.

## Discussion

In this matched cohort study of patients with traumatic injuries in Norway between 2015 and 2018, an increased risk of suicide was observed when comparing patients to matched controls matched on gender and birth year from the general population. After adjusting for potential confounders, psychiatric diagnoses,^3^ preexisting physical health,^24,25,26^ and SEP,^3^ the difference in suicide risk was still higher among patients with traumatic injuries.

Several studies have assessed possible associations between traumatic injury and the risk of suicide. Conner et al^6^ reported an overall RR of 11.1 (95% CI, 8.7-14.1) for suicide among patients with traumatic injuries compared with the general population. When restricting the analysis to only include patients admitted with nonintentional injuries, the corresponding RR was 1.8 (95% CI, 1.0-3.3). A limitation of the study by Conner et al^6^ was that adjustments were only performed for age and gender. In a US study, Ryb et al^7^ reported increased suicide risk following discharge after trauma, with an standard mortality ratio of 1.9 (95% CI, 1.5-2.4). Risk was increased among White patients, males, patients aged 25 to 45 years, and patients influenced by alcohol at the time of injury. Limitations in the study by Ryb et al^7^ were lack of injury-specific data and limited data on psychological and socioeconomic variables. A matched cohort study from Canada by March et al^8^ reported an adjusted odds ratio of 3.7 (95% CI, 2.2-6.0) for suicide and suicidal behavior among patients with traumatic injuries vs controls. March et al^8^ adjusted for SEP and previous psychiatric diagnoses. However March et al^8^ were not only evaluating suicides deaths but also suicidal activity, so the odds ratios presented may not be comparable. These studies^6,7,8^ suggest that traumatic injury is associated with an increased risk of suicide, in line with our findings.

We found Norwegian patients with traumatic injuries were older at the age of suicide compared with controls and females with traumatic injuries had increased risk of suicide compared with the control population. An interpretation of this is that traumatic injury is associated with several risk factors that may lead to suicide. Injury mechanism also was associated with risk of suicide. Exposure to violence was associated with significantly increased risk of suicide, a finding also demonstrated by Herbert et al^27^ in adolescents exposed to violence.^27^ An increased risk of suicide has been reported among patients with cardiac disease and neurological disease and patients treated in the intensive care unit. Studies report that severity of disease was associated with increased risk of suicide.^25,26,28,29^ These findings are in line with our findings that the cumulative incidence of suicide increased with increased severity of injuries. Aasheim et al^30^ reported increased risk of suicide following acute somatic hospitalizations, even in the absence of mental disorders, self-harm, and prior suicide attempts.^30^ An increased risk of suicide has also been observed after injuries, including traumatic brain injury (including mild traumatic brain injury),^29^ isolated pelvic fractures,^31^ spinal injuries,^32^ and osteoporotic fractures.^33^ This suggests that patients who experience life-altering episodes are at increased risk of suicide and that the increased risk of suicide among survivors of trauma may not entirely be related to preinjury behavior and mental health status.

Norway is a high-income country with publicly funded and access to health care and reimbursement of expenses related to medical treatment. These are factors that could potentially reduce the incidence of suicide following traumatic injury. Other countries and health care systems may have different criteria for TTA and, hence, a different cohort of patients. However, the results of this study have generalizability for many high-income countries. Trauma patients are a diverse group of patients who may sustain a wide range of types of injuries. Many medical specialties are involved in treatment, which can result in fragmented follow-up with lack of overall responsibility for managing complex problems, such as reduced physical ability, pain, psychiatric, and social problems.^4^ Finstad et al^4^ have reported that patients with traumatic injuries lacked follow-up after discharge, which may contribute to the increased risk of suicide. The Centers for Disease Control and Prevention report that for every suicide, there may be 30 attempted suicides.^34^ Thus, a significant proportion of patients with traumatic injuries may suffer serious mental and social problems following discharge that may increase suicide risk. We found that many patients with suicide had postdischarge visits with mental health problems, allowing health care professionals an opportunity to possibly intervene. Some health care professionals suggest viewing traumatic injury as a chronic medical condition to facilitate a more holistic approach. For example, hospitals may be reimbursed for providing an outpatient service for patients with traumatic injuries after discharge.^35^

### Strengths and Limitations

A strength of this study is the nationwide and almost full coverage of trauma patients since 2015. Diagnoses of illness were collected from several national registries, increasing the sensitivity of identifying individuals with a disease. Validation of quality of registries has been published previously.^36,37^ Statistics Norway also provides good coverage of data on income, educational level, and other possible confounders for all study participants.

This study has some limitations. One limitation of this study is that included patients could have sustained injuries that led to TTA prior to the study period. Alcohol intoxication has been shown to be associated with the risk of suicide in trauma and is prevalent among patients with traumatic injuries in Norway^7,38^; however no data on alcohol intoxication at the time of injury were available. A possible limitation of this study is the choice of control group. The control group represents the general population in Norway, including individuals who have been hospitalized for other reasons. It is possible that hospitalization for severe illness may be an individual risk factor for suicide, which has not been considered in our analysis. If another control group of other hospitalized patients were compared with patients with traumatic injuries, policymakers could be informed whether trauma patients require more specialized follow-up with respect to suicide risk than other hospitalized patients. Another limitation of this study is that to have a conservative estimate of the risk, we chose to start the observation period 2 weeks after discharge, which potentially can lead to a lower estimate of the risk.

## Conclusions

In this matched cohort study of patients with traumatic injuries registered in a nationwide registry, we found a 9-fold increased risk of suicide after 2 years compared with general-population controls matched on gender and birth year. The risk was also found to be significantly increased after adjusting for preexisting mental health problems, comorbidities, and socioeconomic factors. The results of this study suggest that patients with traumatic injuries should receive more comprehensive follow-up after discharge to reduce the risk of death by suicide.

## Notes

Supplement 1. **eFigure 1.** Directed acyclic graph **eFigure 2.** Cumulative incidence of suicide **eTable 1.** Baseline demographic data for trauma patients in the study population **eTable 2.** Baseline demographic data for suicide deaths in the study population

Supplement 2.
Data Sharing Statement
