# Supplementary material for: Risk of Suicide in Patients With Traumatic Injuries
Source: JAMA Netw Open. 2026 Jan 15;9(1):e2554168. doi: 10.1001/jamanetworkopen.2025.54168 (PMC12809368; doi:10.1001/jamanetworkopen.2025.54168)
Supplement: Supplement 1. — eFigure 1. Directed acyclic graph eFigure 2. Cumulative incidence of suicide eTable 1. Baseline demographic data for trauma patients in the study population eTable 2. Baseline demographic data for suicide deaths in the study population [file jamanetwopen-e2554168-s001.pdf]

Supplemental Online Content

Rasmussen A, Nordseth T, Stenehjem JS, Gran JM, Lien L, Rosseland LA. Risk of suicide in patients with traumatic injuries. *JAMA Netw Open*. 2026;9(1):e2554168. doi:10.1001/jamanetworkopen.2025.54168

eFigure 1. Directed acyclic graph

eFigure 2. Cumulative incidence of suicide

eTable 1. Baseline demographic data for trauma patients in the study population

eTable 2. Baseline demographic data for suicide deaths in the study population

This supplemental material has been provided by the authors to give readers additional information about their work.

© 2025 Rasmussen A et al. *JAMA Network Open*.

eFigure 1: Directed Acyclic Graph (DAG). Traumatic injury as exposure and suicide as an outcome. Risk factors and associations for suicide are presented.

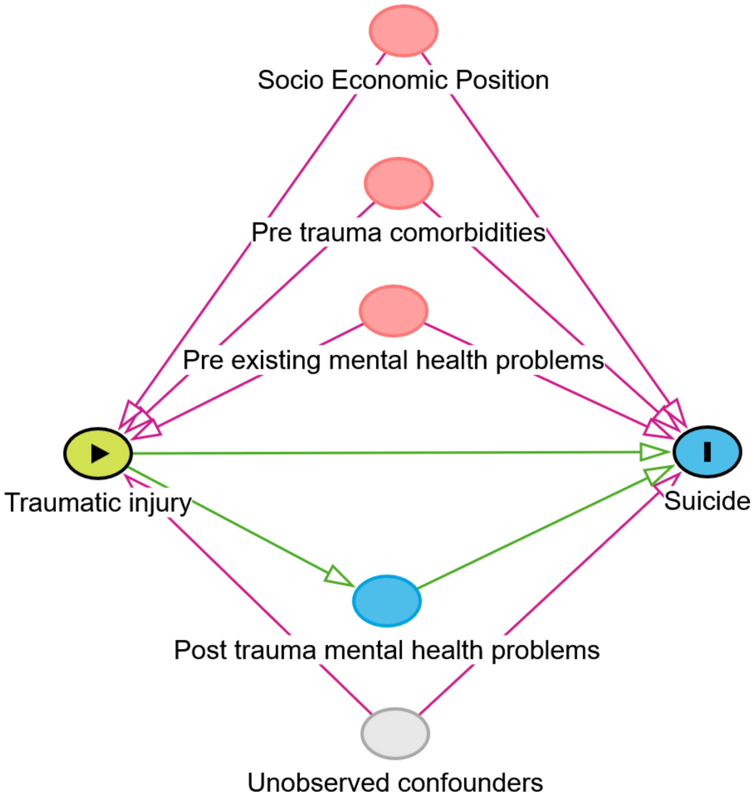

© 2025 Rasmussen A et al. *JAMA Network Open*.

eFigure 2: Cumulative incidence of suicide. Stratified on A) Gender, B) Psychiatric diagnoses after trauma event, C) Marital status, D) Income (USD), E) Highest education

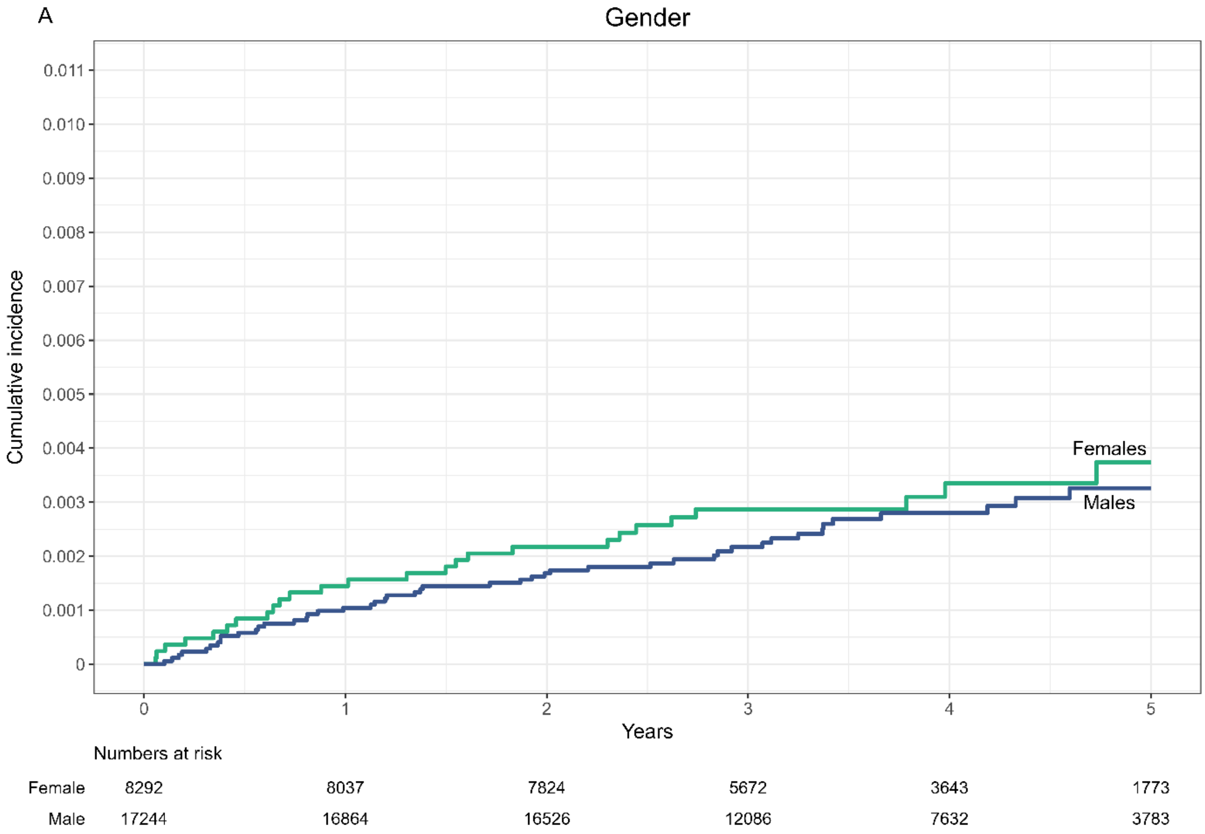

© 2025 Rasmussen A et al. *JAMA Network Open*.

B

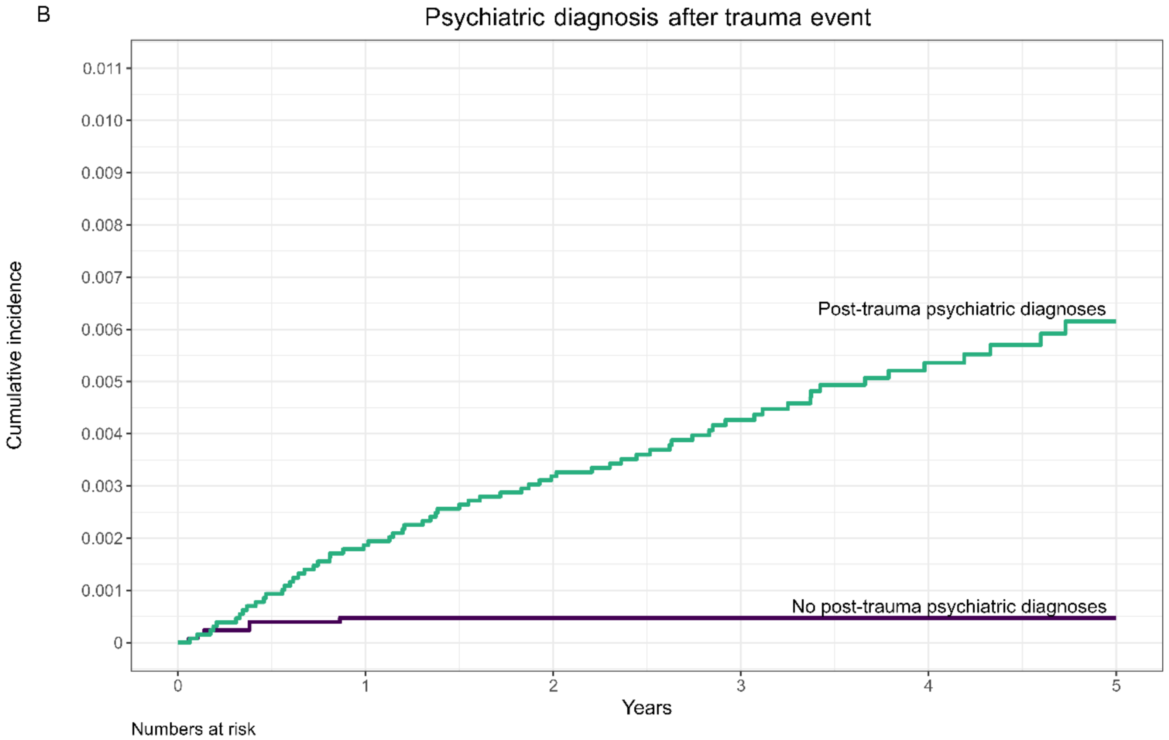

Numbers at risk

No diagnosis	12667	12354	12135	8595	5251	2511
Diagnosis	12869	12547	12215	9163	6024	3045

C

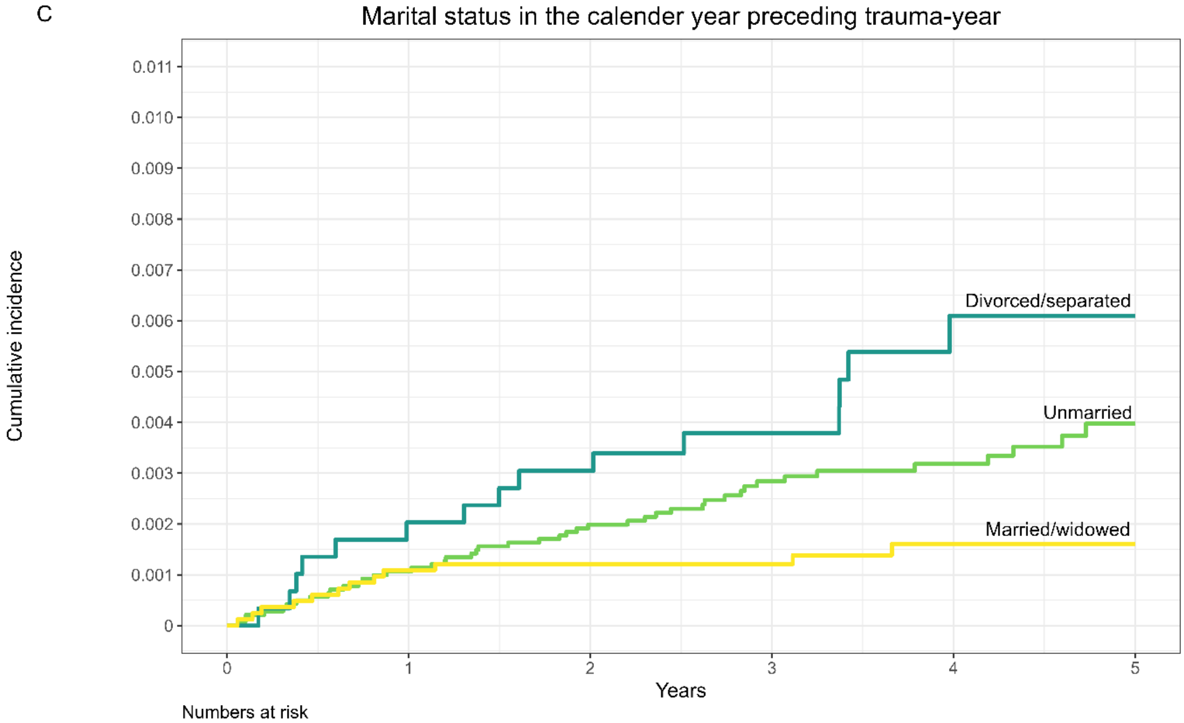

Numbers at risk

Unmarried	14087	13975	13794	10167	6539	3262
Divorced/separated	2955	2851	2765	2001	1250	616
Married/Widowed	8243	7825	7545	5396	3352	1615

© 2025 Rasmussen A et al. *JAMA Network Open*.

D

Income (USD) in the calender year preceding trauma-year

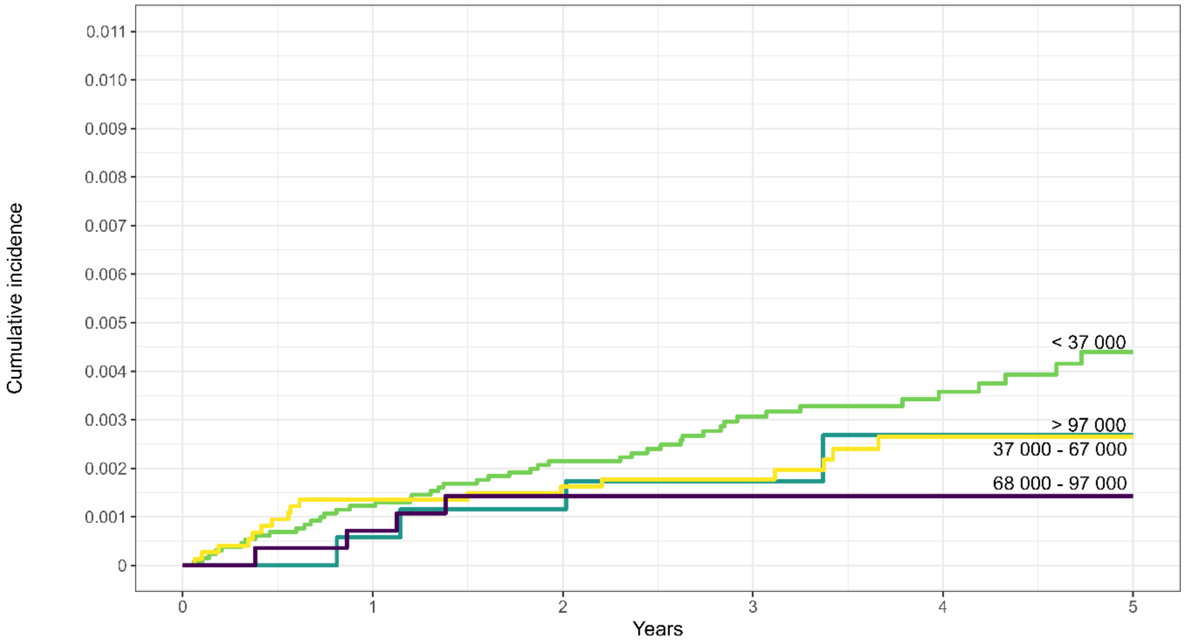

Numbers at risk

	0	1	2	3	4	5
<37000	13056	12709	12415	9205	5969	2976
>97000	1732	1714	1695	1208	731	349
37000-67000	7374	7136	6951	4999	3119	1543
68000-97000	2798	2770	2729	1942	1195	572

E

Highest level of education

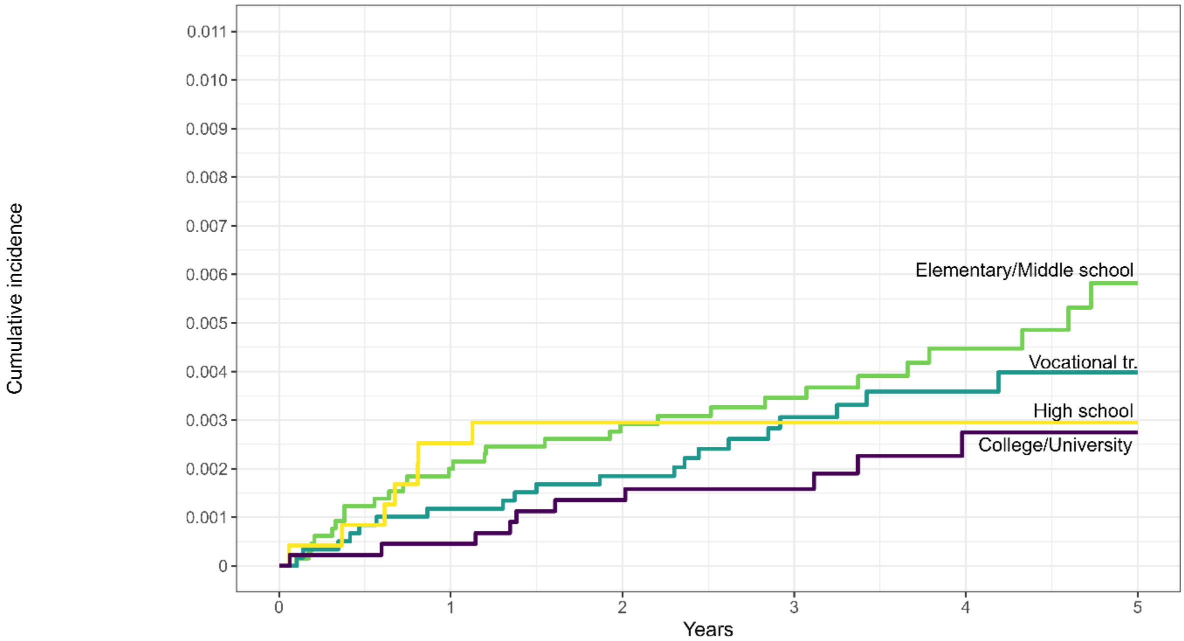

Numbers at risk

	0	1	2	3	4	5
Elementary / Middle school	6510	6255	6078	4413	2774	1406
Vocational training	5953	5846	5750	4167	2631	1287
High school	2375	2204	2082	1499	945	461
College / University	4437	4345	4246	3050	1914	928

© 2025 Rasmussen A et al. *JAMA Network Open*.

eTable 1: Baseline demographic data for trauma patients in the study population.

Characteristic	**No Event**, N = 25,464	**Suicide**[b], N = 72	p-value[a]
**Gender (M/F), n (%)**			0.51
F	8,266 (32)	26 (36)	
M	17,198 (68)	46 (64)	
**Age (years), Mean (SD)**	41 (23)	42 (19)	0.62
**Charlson Comorbidity Index, n (%)**			0.46
0	16,811 (66)	49 (68)	
1	3,967 (16)	9 (12)	
2	2,149 (8)	5 (7)	
3	1,021 (4)	<5 (<5)	
4	558 (2)	<5 (<5)	
5	298 (1)	<5 (<5)	
>=6	660 (3)	<5 (<5)	
**Mechanism of injury, n (%)**			<0.001
Traffic	11,752 (47)	18 (27)	
Violence	3,410 (14)	24 (36)	
Falls	8,910 (36)	20 (30)	
Other mechanisms	745 (3)	5 (8)	
Missing data	647	5	
**Injury Severity Score categories, n (%)**			<0.001
1-8 Minor	16,077 (63)	36 (50)	
9-15 Moderate	6,013 (24)	14 (19)	
16-24 Severe	2,131 (8)	9 (12)	
>=25 Very severe	1,243 (5)	13 (18)	
**Educational Level, n (%)**			0.01
Age less than 20 years	5,749 (13)	6 (8)	

© 2025 Rasmussen A et al. *JAMA Network Open*.

Characteristic	**No Event**, N = 25,464	**Suicide**[b], N = 72	p-value[a]
Elementary / Middle school	6,481 (26)	29 (40)	
High School	2,368 (10)	7 (10)	
Vocational training	5,933 (24)	20 (28)	
College / University	4,429 (18)	10 (14)	
Missing data	506	0	
**Income group (USD), n (%)**			0.20
<37 000	13,010 (52)	46 (65)	
37 000 - 67 000	7,357 (30)	17 (24)	
68 000 – 97 000	2,794 (11)	<5 (<6)	
>97 000	1,728 (7)	<5 (<6)	
Missing data	575	1	
**Marital Status, n (%)**			0.007
Divorced/separated	2,940 (12)	15 (21)	
Married/Widowed	8,231 (32)	12 (17)	
Unmarried	14,042 (55)	45 (62)	
Missing data	251 (1)	0 (0)	
**Any pre-traumatic ICD-10 F-diagnosis or ICPC-2 P-diagnosis, n (%)**	9,694 (38)	57 (79)	<0.001
**Any pre-traumatic ICD 10 F-diagnosis, n (%)**	4,610 (18)	50 (69)	<0.001
**Any post-traumatic ICD-10 F-diagnosis or ICPC-2 P-diagnosis, n (%)**	12,803 (50)	66 (92)	<0.001

[a]Pearson's Chi-squared test; Welch Two Sample t-test

[b]Data Protection Officer policy is not to report numbers less than five

© 2025 Rasmussen A et al. *JAMA Network Open*.

eTable 2: Baseline demographic data for suicides in the study population.

Characteristic	Controls[b], N = 94	Trauma patients[b], N = 72	p-value[a]
**Gender (M/F), n (%)**			0.005
F	16 (17)	26 (36)	
M	78 (83)	46 (64)	
**Age (years), Mean (SD)**	36 (17)	43 (19)	0.029
**Charlson Comorbidity Index, n (%)**			0.42
0	74 (79)	49 (68)	
1	10 (11)	9 (12)	
2	5 (5)	5 (7)	
3	<5 (<5)	<5 (<5)	
4	0 (0)	<5 (<5)	
5	0 (0)	<5 (<5)	
>=6	<5 (<5)	<5 (<5)	
**Income group (1000 USD), n (%)**			0.44
<36	48 (53)	46 (65)	
37-67	29 (32)	17 (24)	
68-97	9 (10)	<5 (<6)	
>97	5 (6)	<5 (<6)	
Missing data	<5	<5	
**Educational Level, n (%)**			0.065
Age under 20 years	5 (5)	<5 (<5)	
Elementary school	45 (49)	33 (46)	
High School	<5 (<5)	7 (10)	
Vocational training	18 (20)	20 (28)	
College / University	20 (22)	10 (14)	
Missing Data	<5	0	

© 2025 Rasmussen A et al. *JAMA Network Open*.

Characteristic	Controls[b], N = 94	Trauma patients[b], N = 72	p-value[a]
**Marital Status, n (%)**			0.11
Divorced/separated	11 (12)	15 (21)	
Married/Widowed	10 (11)	12 (17)	
Unmarried	71 (76)	45 (62)	
Missing data	<5 (<5)	0 (0)	
**Any pre-index ICD-10 F-diagnosis or ICPC-2 P-diagnosis, n (%)**	60 (64)	57 (79)	0.032
**Any pre-index ICD 10 F-diagnosis, n (%)**	33 (35)	50 (69)	<0.001
**Any post-index ICD-10 F-diagnosis or ICPC-2 P-diagnosis, n (%)**	75 (80)	66 (92)	0.034

[a]Pearson's Chi-squared test; Welch Two Sample t-test

[b]Data Protection Officer policy is not to report numbers less than five

© 2025 Rasmussen A et al. *JAMA Network Open*.
