# Supplementary material for: Risk of Suicide in Patients With Traumatic Injuries
Source: JAMA Netw Open. 2026 Jan 15;9(1):e2554168. doi: 10.1001/jamanetworkopen.2025.54168 (PMC12809368; doi:10.1001/jamanetworkopen.2025.54168)
Supplement: Supplement 2. — Data Sharing Statement [file jamanetwopen-e2554168-s002.pdf]

Data Sharing Statement

Rasmussen. Risk of Suicide in Patients With Traumatic Injuries. *JAMA Netw Open*. Published January 15, 2026. doi:10.1001/jamanetworkopen.2025.54168

Data
Data available: Yes
Data types: Deidentified participant data
How to access data: Deidentified data can be obtained after application to the project leader Leiv Arne Rosseland.
When available: With publication

Supporting Documents
Document types: None

Additional Information
Who can access the data: Researchers whose proposed use of the data has been approved
Types of analyses: any pourpose approved by the project leader
Mechanisms of data availability: after approval of a proposal
